# Supplementary material for: Targeting KRAS Regulation with PolyPurine Reverse Hoogsteen Oligonucleotides
Source: Int J Mol Sci. 2022 Feb 14;23(4):2097. doi: 10.3390/ijms23042097 (PMC8876201; doi:10.3390/ijms23042097)
Supplement: Supplementary file 1 [file ijms-23-02097-s001.zip › ijms-1561501-supplementary.pdf]

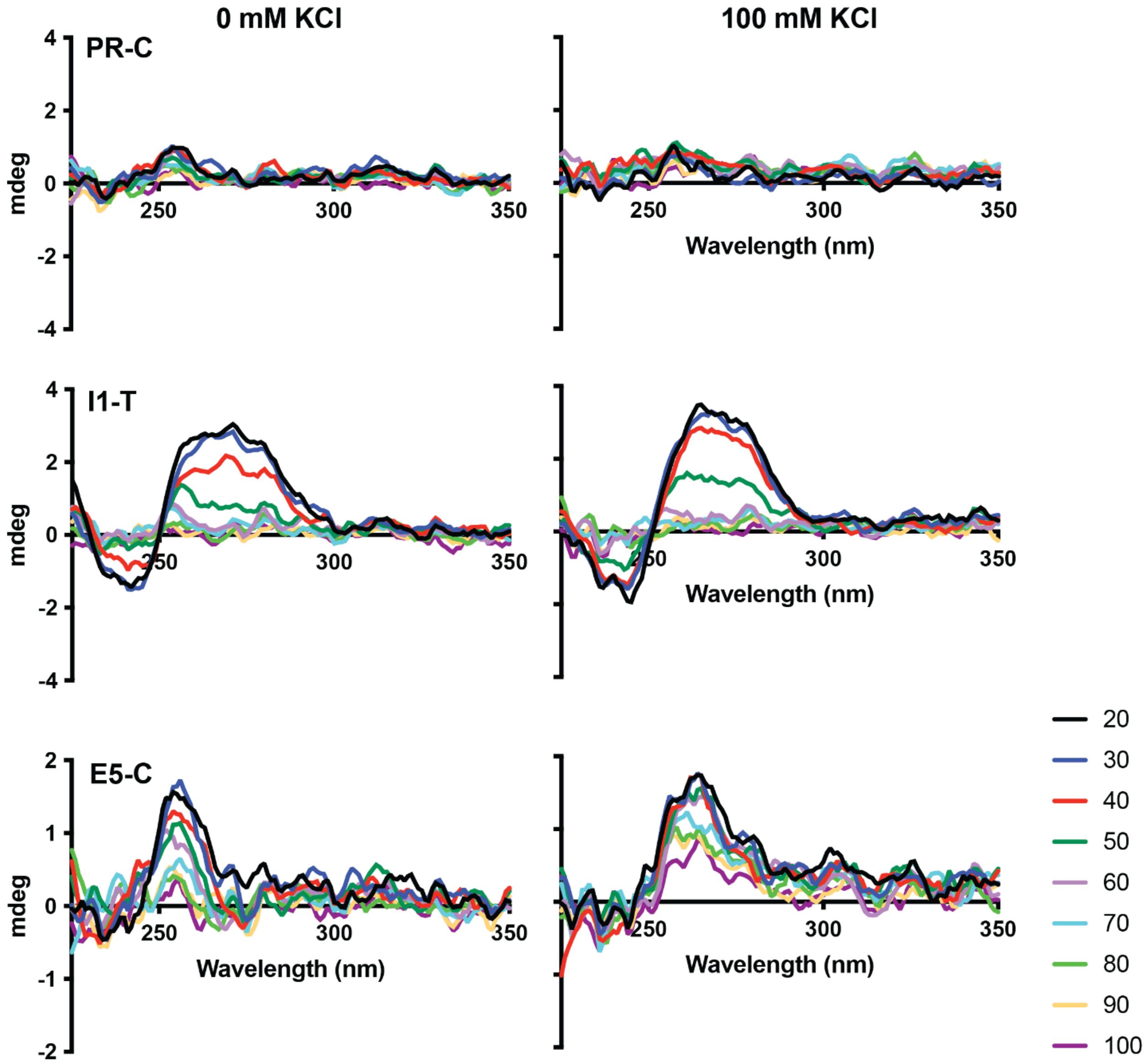

Figure S1. ECD Spectra for three novel G4FS as a function of temperature. PR-C formed no discernible secondary DNA structure at any temperature in either the absence (left) or presence (right) of 100 mM KCl (top). I1-T formed hairpin structures with comparable thermal stability in the absence or presence of KCl (middle), and E5-C demonstrated an induction of the G4 sequence, as evidenced by a right shift in spectral maxima towards 262 nm, with the addition of KCl, and also an increase in thermal stability.

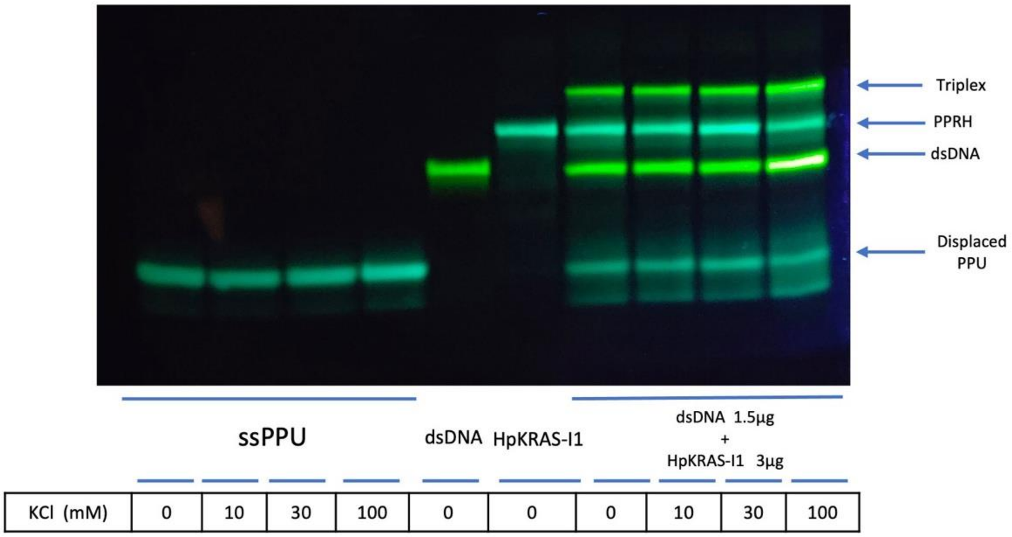

Figure S2. Displacement analysis of the Polypurine (PPU) strand in Intron 1 probe with increasing concentrations of KCl. Bindings were performed using 1.5 µg of dsDNA labelled with FAM (green) in the polypyrimidine (PPY) strand only, then incubated as described in M&M with 3µg of Hp-KRAS-I1. The mobility of the ssPPU, dsDNA and PPRH was checked by loading 1.5µg in the corresponding tracks. The resulting structures were resolved by native polyacrylamide (12%) gel electrophoresis. PPRHs, ssPPU and displaced PPU were visualized after Thioflavin-T staining (cyan bands).

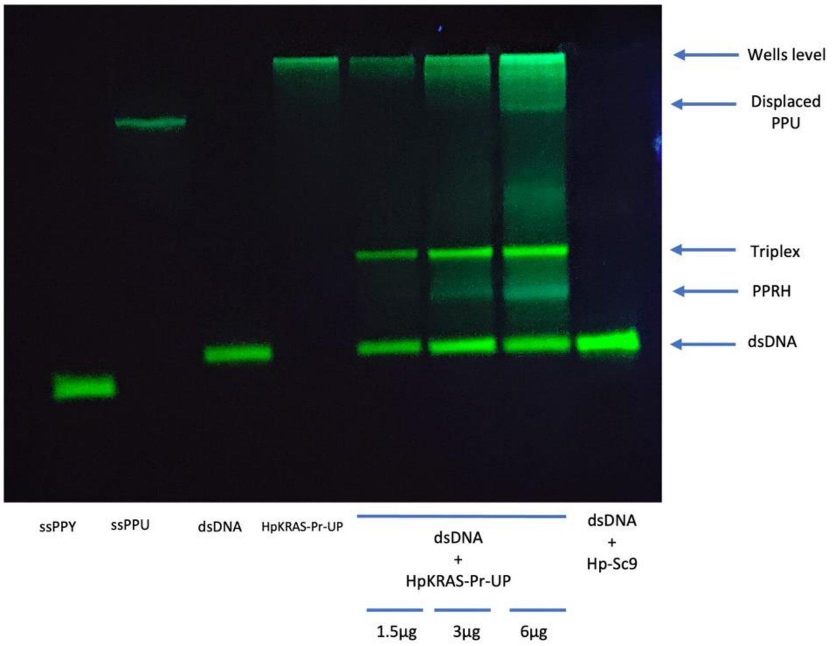

Figure S3. Displacement analysis of the Polypurine (PPU) strand in the distal promoter probe. Bindings were performed using 1.5 µg of dsDNA labelled with FAM (green) in the polypyrimidine (PPY) strand only, then incubated as described in M&M with the indicated amounts of Hp-KRAS-Pr-Up PPRH or 1.5 µg of the negative control HpSc9. The resulting structures were resolved by native polyacrylamide (12%) gel electrophoresis. PPRHs, ssPPU and displaced PPU were visualized after Thioflavin-T staining (cyan bands).

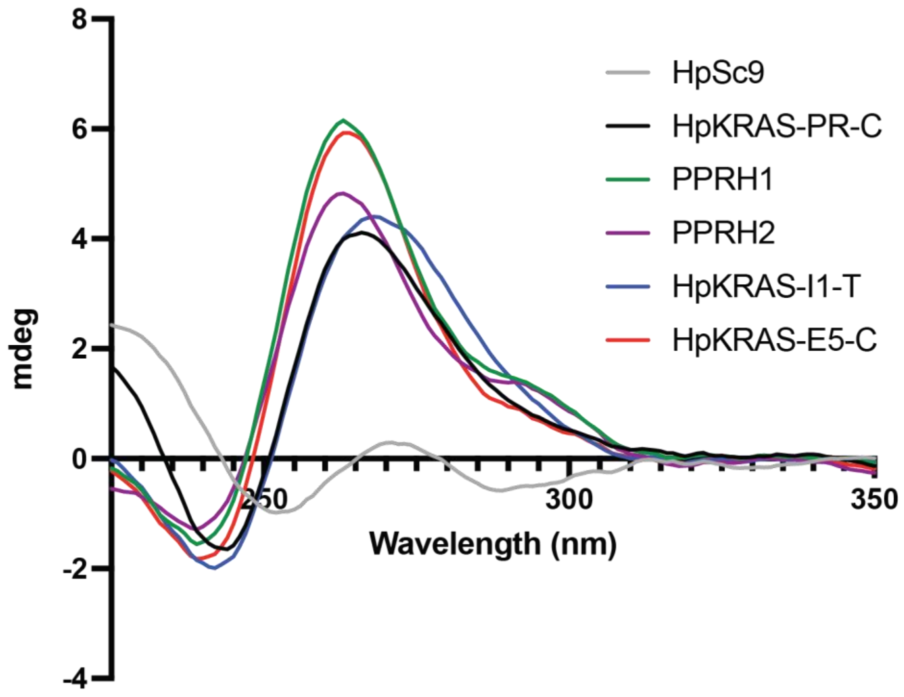

Figure S4. ECD spectra of PPRH sequences. Spectra were recorded from 225-350 nM at room temperature for all PPRH sequences in the presence of 100 mM KCl.
